# Risk of SARS-CoV-2 Reinfection 3 Years after the Start of the Pandemic: A Population-Level Observational Study

**DOI:** 10.3390/life13112111

**Published:** 2023-10-24

**Authors:** Cecilia Acuti Martellucci, Maria Elena Flacco, Graziella Soldato, Giuseppe Di Martino, Roberto Carota, Annalisa Rosso, Marco De Benedictis, Graziano Di Marco, Rossano Di Luzio, Francesco Lisbona, Antonio Caponetti, Lamberto Manzoli

**Affiliations:** 1 Department of Environmental and Prevention Sciences, University of Ferrara, 44121 Ferrara, Italy; cecilia.martellucci@unife.it (C.A.M.); mariaelena.flacco@unife.it (M.E.F.); annalisa.rosso@unife.it (A.R.); 2 Local Health Unit of Pescara, 65124 Pescara, Italy; graziella.soldato@ausl.pe.it (G.S.); giuseppe.dimartino@ausl.pe.it (G.D.M.); roberto.carota@ausl.pe.it (R.C.); marco.debenedictis@ausl.pe.it (M.D.B.); graziano.dimarco@ausl.pe.it (G.D.M.); rossano.diluzio@ausl.pe.it (R.D.L.); antonio.caponetti@ausl.pe.it (A.C.); 3 Department of Medical and Surgical Sciences, University of Bologna, 40100 Bologna, Italy; francesco.lisbona@studio.unibo.it

**Keywords:** SARS-CoV-2, COVID-19, reinfection, case fatality rate, vaccination

## Abstract

The risk of SARS-CoV-2 reinfections changes as new variants emerge, but the follow-up time for most of the available evidence is shorter than two years. This study evaluated SARS-CoV-2 reinfection rates in the total population of an Italian province up to three years since the pandemic’s start. This retrospective cohort study used official National Healthcare System data on SARS-CoV-2 testing and vaccinations, demographics, and hospitalizations in the Province of Pescara, Italy, from 2 March 2020 to 31 December 2022. A total of 6541 (5.4%) reinfections and 33 severe and 18 lethal COVID-19 cases were recorded among the 121,412 subjects who recovered from a primary infection. There were no severe events following reinfection in the young population, whereas 1.1% of reinfected elderly died. A significantly higher reinfection risk was observed among females; unvaccinated individuals; adults (30–59 y); and subjects with hypertension, COPD, and kidney disease. Up to three years after a primary SARS-CoV-2 infection, the majority of the population did not experience a reinfection. The risk of severe COVID-19 following a reinfection was very low for young and adult individuals but still high for the elderly. The subjects with hybrid immunity showed a lower reinfection risk than the unvaccinated.

## 1. Introduction

In order to explore the degree of protection conferred by natural or hybrid immunity, a number of studies estimated the rate of reinfection after recovery from a primary SARS-CoV-2 infection [1,2]. These rates largely varied by geographical area [3], vaccination status [4], and predominant virus variant [5,6]. Indeed, variants evolve over time, and continuous monitoring is necessary to obtain updated estimates of the reinfection risk of the population [7,8]. Indeed, the evaluation of the epidemiological and clinical characteristics of SARS-CoV-2 reinfections is pivotal for modeling future trends of the pandemic and organizing the relative public health response [9] as well as examining the immunological mechanisms potentially involved in the progressive waning of both natural and hybrid immunity [8,10].

Most of the available evidence is based on follow-up periods shorter than 24 months [11,12], and the few studies with longer follow-ups relied on self-reported infections [13] or on testing performed in single healthcare facilities [14]. We carried out a retrospective cohort analysis of the entire population of an Italian province and estimated the rate of SARS-CoV-2 infections after three years from the start of the pandemic.

## 2. Materials and Methods

This retrospective cohort study expands two earlier assessments with the addition of one year of follow-up [15,16]. The study population included all citizens residing permanently or temporarily in the Italian Province of Pescara on 1 January 2020, aged 10 years or older, and with ≥1 positive nasopharyngeal swab detected via RT-PCR by the regional official laboratories or via rapid antigen test by local pharmacies (the latter only since January 2021).

Data collection started at the beginning of the pandemic (2 March 2020) and continued to 31 December 2022. All data were obtained from the following official National Healthcare System databases, which are compiled, updated, and shared with the Italian Institute of Health on a daily basis [17]:

- COVID-19 vaccines (from 1 January 2021—start of the vaccination campaign—to 31 December 2022);
- SARS-CoV-2 laboratory or pharmacy assays and confirmed COVID-19 cases (up to 15 February 2023 allowing for at least 45 days of follow-up after immunization);
- Demographic (Italian “Anagrafica” up to 15 February 2023).

In order to merge the data from the sources listed above, deterministic linkage was performed using an encrypted fiscal code (as all databases included univocal fiscal codes generated on the basis of the high-quality National Tax Registry).

A reinfection episode occurred when two positive swabs were detected at least 45 days apart provided that at least one negative swab had been detected between the two positive ones [15,18]. To be defined as severe COVID-19, cases had to be confirmed virologically, diagnosed by a specialist physician, and also require hospital admission. COVID-19-related deaths were defined as a severe COVID-19 infection causing death within 60 days from diagnosis [19].

Vaccinated individuals were those that had received at least one dose of BNT162b2, ChAdOx1 nCoV-19, mRNA-1273, NVX-CoV2373, or JNJ-78436735 vaccines at least 14 days before reinfection. The follow-up was set to begin on the day of the first positive test and to end on the first day of reinfection; alternatively, it was censored on February 15, 2023.

To evaluate the potential association between reinfection and selected potential risk factors, including diabetes, hypertension, major cardiovascular or cerebrovascular diseases (CVD), chronic obstructive pulmonary diseases (COPD), kidney disease, and cancer, we also extracted the data of the following official regional datasets: (a) COVID-19 cases, (b) co-pay exemption (Italian “Esenzioni Ticket”), and (c) administrative discharge abstracts from the previous ten years. In the last dataset, the following ICD-9-CM codes in any diagnosis field were used: 250.xx (diabetes); 401.xx–405.xx (hypertension); 410.xx–412.xx, 414.xx–415.xx, 428.xx, or 433.xx–436.xx (CVD); 491.xx–493.xx (COPD); 580.xx–589.xx (kidney disease); and 140.xx–172.xx or 174.xx–208.xx (cancer).

The reinfection rate was calculated for the overall sample and according to age, gender, the above risk factors, and time after primary infection (1.5–5, 6–12, 13–18, 19–24, and 24–35 months). The relative hazards of reinfection were computed using the Cox proportional hazard analysis, adjusted a priori for age, gender, vaccination status (no doses, up to two, and three or more), severe COVID-19 during first infection, and selected potential risk factors. In order to verify the validity of the proportional hazard assumption, Schoenfeld’s test was used. For all reinfections, we also calculated the proportions of severe and lethal COVID-19. The main analyses were also repeated twice, separately considering the pre-Omicron predominance period (approximately from the start of the follow-up to 31 December 2021) and the Omicron predominance period (approximately from 1 January 2022 to the end of follow-up) [20]. The analyses referring to the pre-Omicron period were restricted to the subjects who had a positive swab before 17 November 2021 (to account for a minimum of 45 days between the first and second infection), and the subjects who had a second positive swab after 31 December 2021 were reassigned to the group without a reinfection. The analyses referring to the Omicron period were restricted to the subjects that did not have a reinfection before 1 January 2022. All tests were two-sided, and statistical significance was indicated by a *p*-value < 0.05. The analyses were performed using Stata 13.1 (Stata Corp., College Station, TX, USA, 2014).

The study protocol was approved by the Ethics Committee of the Emilia-Romagna Region (code 287, approved on 24 March 2020).

## 3. Results

**Figure 1:**
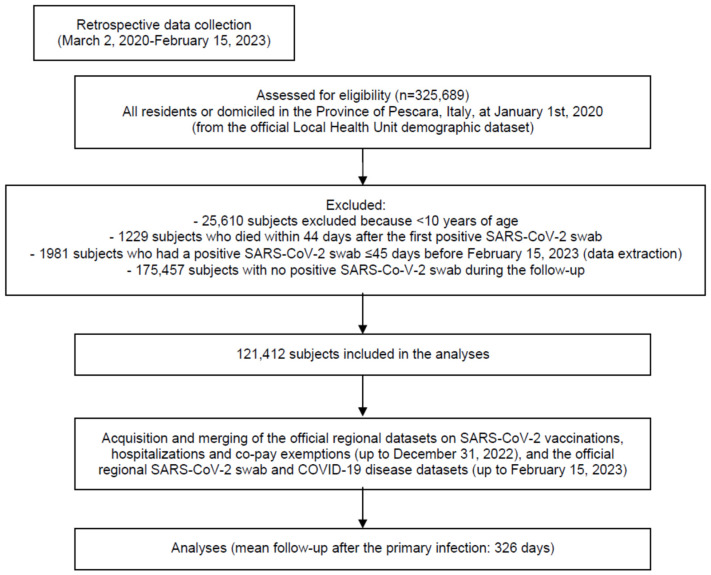
Study flowchart in line with the STROBE (Strengthening the Reporting of Observational Studies in Epidemiology) statement (http://www.strobestatement.org (accessed on 22 May 2023)).

We included a total of 121,412 subjects who recovered from a primary infection (Figure 1). During the follow-up (which lasted 326 days on average; standard deviation—SD—191 days), 6541 reinfections were recorded (5.39% of the sample). Among the reinfected subjects, 51 had a severe (*n* = 33) or lethal (*n* = 18) COVID-19 infection (0.8% of the reinfected population; 0.4‰ of the infected population). None of the 51 severe cases following reinfection were recorded among subjects younger than 40 years, and all of the 18 deceased individuals were older than 60 years (17 were older than 70 years). Overall, among the 1579 reinfected elderly (≥60 y), the rates of severe and lethal COVID-19 were 2.1% and 1.1%, respectively (1.1‰ and 0.6‰, respectively, of the 28,761 infected elderly population).

As shown in Table 1, the mean time elapsed from the first infection to reinfection was 518 days (SD: 204 days). From the time of the primary infection, the rate of reinfections showed a fairly constant growth, reaching a peak of 7.34% in months 19–24.

The rate of reinfection significantly varied by age, being lowest among individuals aged 10–29 years (3.70%) and highest among subjects aged 30–59 years (6.17%—Table 2). Multivariable analyses, adjusting for vaccination and other covariates, confirmed that the individuals 30–59 y old carried the highest risk of reinfection, but younger populations showed a higher probability than the elderly of experiencing a second infection (both *p* < 0.005).

The other independent, significant predictors of a higher reinfection risk were female gender (adjusted HR: 1.37; 95% Confidence Interval: 1.30–1.44), hypertension (1.15; 1.04–1.27), COPD (1.14; 1.02–1.27), kidney disease (1.23; 1.03–1.47), and the presence of at least one comorbidity (1.13; 1.05–1.20—Table 2). In contrast, hybrid immunity was associated with a significantly lower likelihood of reinfection: the highest rate was observed among the unvaccinated (11.2%) and the lowest among those who received at least one booster dose (2.52%). According to the multivariable analysis, compared to the unvaccinated, all vaccinated individuals showed a 30% decrease in the risk of reinfection (*p* < 0.001).

The analyses were repeated by stratifying by epidemic wave, and the reinfections occurring before or during the Omicron predominance period were analyzed separately (Supplementary Tables S1 and S2). As the vast majority of the reinfections were recorded during the Omicron wave (99.1%—*n* = 6481), the analyses restricted to this period yielded results that were identical to the overall sample (Table S2). Given that only 60 reinfections were observed during the initial, pre-Omicron wave, the analyses restricted to that period lacked statistical power, and only a younger age (HR: 5.21; 95% CI: 1.65–16.5) and vaccination (0.39; 0.16–0.99) were significantly associated with reinfection (Table S1).

## 4. Discussion

In the present cohort study on the total population of one Italian province followed for up to three years, SARS-CoV-2 reinfections were relatively uncommon, although their rate increased over time, especially during the predominance of the Omicron and more contagious variants [21]. Arguably, as a consequence of both natural immunity and vaccination, more than 90% of the population did not experience a second infection after the first episode. Furthermore, less than 1 out of 1000 individuals who recovered from a primary infection experienced a reinfection that led to severe COVID-19 disease.

A large meta-analysis including articles published until 30 June 2022 found a SARS-CoV-2 reinfection rate of about 1% [5]. However, a higher rate was somewhat inevitable given the longer follow-up of the present study. Indeed, more recent evidence from the United Kingdom showed a reinfection rate of approximately 10% [13]. This figure is consistent with other studies, which also suggest that Omicron variants determined a higher reinfection rate compared to previous variants [22,23,24,25,26]. Furthermore, data from the year 2022 in the USA show that reinfection rates could reach up to 29% during an Omicron BQ.1/BQ.1.1 wave [11]; however, this variant only became predominant in Italy after the conclusion of the present follow-up period [27]. Such growing infectivity appears to be driven by a highly efficient immune evasion (in response to antibody pressure) together with changes in the ACE2 binding receptor [27,28]. Data derived from long follow-ups are especially informative to determine the potential impact of the evolving variants and evaluate the policy shift towards co-existence with the virus. Although our findings suggest that natural and hybrid immunity could last for up to three years in the majority of individuals, we could not assess the potential effects of the most recent and contagious BQ.1/BQ.1.1 variants.

Various studies showed a reduced severity of reinfections compared to primary infections, although rarely as low as the ≈1% found in this cohort [29,30,31]. In line with our findings, however, a meta-analysis of studies published until 11 December 2022 reported a prevalence of severe disease lower than 1% after a reinfection [29]. Also, compared with SARS-CoV-2 primary infection cases, the risk of severe illness was reduced by 86%, although the authors reported that these estimates might be greatly influenced by variations in hospital capacity and testing policies across subsequent epidemic waves [29,30]. In addition, in this as in previous reports [31,32], the risk of severe outcomes following a reinfection widely varied by age: while reinfection did not seem to be a threat for the youngest, among the reinfected elderly, the risk of serious or lethal COVID-19 remained high (>3%).

Besides the potential predictors of a more severe outcome, it must be taken into account that in this as in all studies without updated seroprevalence data, the risk of reinfection morbidity and lethality can be overestimated as a result of an underestimation of the reinfection prevalence [33]. However, the Italian policies adopted during the pandemic—according to which the subjects with a reported infection were granted access to work or public facilities through the so-called “Green Pass” [34]—may have limited the amount of unreported infections, and indeed the rates of severe outcomes observed in this study are among the lowest recorded so far. In any case, further studies including seroprevalence data are definitely needed to provide more precise estimates of the risk of serious outcomes following SARS-CoV-2 reinfection.

With regard to the impact of COVID-19 vaccination, in this population, all vaccinated subjects carried a significantly lower risk of reinfection compared to the unvaccinated. This finding is consistent with three systematic reviews, which documented a superior protection of hybrid over natural immunity not only against reinfection but also against severe disease and hospitalization following reinfection [4,26,35]. One recent large cohort study of healthcare workers is in accordance as well [36]. Unexpectedly, however, in our sample, the individuals who received three or more doses did not show a lower reinfection rate than those who received only one or two doses. Such an equivalence might have been determined by the specific restrictions that were in place in Italy during most of the pandemic period: given that only the individuals who received at least three vaccine doses were allowed to access work or public spaces, these persons were inevitably much more likely to be exposed to the virus than those who received a lower number of doses [34]. Another Italian study found no effect of vaccination on the likelihood of reinfection [37]. However, that study had a shorter follow-up, used a 90-day cut-off to define reinfections, selected matched cohorts, and was made in an area with almost double the population density of the Province of Pescara, all factors that hinder a direct comparison.

In agreement with previous research [13,38], age, gender, and some comorbidities were found to be independently associated with the likelihood of reinfection. Specifically, a higher reinfection risk was observed among females, which could reasonably be explained by a more pronounced test-seeking behavior in women [13,16,31]. The age group ranging between 30 to 59 years showed the highest infection rate, with younger people at intermediate risk and older ones at the lowest risk. This U-shaped effect has been attributed to the wider intra- and inter-generational interactions of adults, which are therefore at the highest risk of infection [13,16,31,39]. Finally, the increased reinfection risk observed for people with specific comorbidities has been linked to the suboptimal physical and immunological status of these individuals, which was among the reasons to prioritize high-risk groups during the vaccination campaign [31,35].

Regarding the potential policy implications of the present findings, one important point concerns the duration of the hybrid (and, on a lesser extent, the natural) immunity against the most severe evolution of reinfections, which is expected to be high up to three years after the primary infection. Thus, current vaccination policies should clearly prioritize subjects who have not been vaccinated and/or infected so far. In addition, as the risk of morbidity and mortality due to reinfection remains sizeable in elderly individuals and among those with hypertension, COPD, and kidney disease, these subsets of the population could be prioritized for vaccination regardless of the vaccination/infection status and should be closely monitored if reinfected.

This study used the longest follow-up to date and comprehensively evaluated official data from the whole population of one Italian province, thus reducing the selection and classification biases that may arise from retrospectively identifying matched cohorts or using only tests performed by one institution [14,37]. Also, we used the official definition of reinfection provided by the US Centers for Disease Control and Prevention (CDC) [18], ensuring comparability across studies [40,41] and with previous evaluations of the same population [16]. The study also presents limitations, which should be considered. First, in addition to the above-mentioned lack of seroprevalence data and the impossibility to evaluate the impact of the Omicron BQ.1/BQ.1.1 variants, the adopted CDC definition might have resulted in losing a small quota of reinfections in the case of a missing negative test between two positive ones as well as in partial comparability with studies which used different definitions of reinfection (e.g., 60 or 90 days after the first infection or absence of an intermediate negative test). Second, extended genotyping data were not available, which prevented us from exploring the contribution of specific variants. Finally, residual confounding could be derived from unmeasured characteristics such as severity, duration, and drug therapy for chronic conditions but also professional exposure to SARS-CoV-2, student status, social activities, propensity to self-isolate, and all the other behaviors which may influence the risk of infection or the likelihood of getting tested [13].

## 5. Conclusions

Up to three years after a primary SARS-CoV-2 infection, the analysis of all the residents of one Italian province confirmed that SARS-CoV-2 reinfections are becoming more frequent over time, although the overall rate remained below 10%. Although the reinfections did not cause any serious disease among the youngest, the risk of severe or lethal COVID-19 among the reinfected elderly was high. A significantly higher risk of reinfection was observed among the unvaccinated; females; and subjects with hypertension, COPD, and kidney disease. Further studies are required to estimate the risk of reinfection during the Omicron BQ.1/BQ.1.1 waves as well as to provide more precise estimates of infection fatality rates using seroprevalence data.

## Figures and Tables

**Table 1:** Number of SARS-CoV-2 reinfections according to time after the first infection.

	Total Sample	Reinfections ^a^	
	(*n* = 121,412)	(*n* = 6541)	
Mean time after the primary infection, days (SD)	326 (191)	518 (204)	--
Months since first infection	***n***	% (*n*)	**95% CI (%)**
- Less than 6 (<183 days)	121,412	0.84 (1019)	(0.79–0.89)
- 6–12 (183–365 days)	101,446 ^b^	3.12 (3165)	(3.01–3.23)
- 13–18 (366–548 days)	44,235 ^c^	2.05 (905)	(1.92–2.18)
- 19–24 (549–730 days)	15,322 ^d^	7.34 (1125)	(6.93–7.77)
- 24+ (731–1076 days)	8555 ^e^	3.82 (327)	(3.43–4.25)

^a^ Two positive tests detected at least 45 days apart with at least one negative test detected between the first and second episode. ^b^ The uninfected subjects with a follow-up shorter than 183 days have been excluded. ^c^ The uninfected subjects with a follow-up shorter than 366 days have been excluded. ^d^ The uninfected subjects with a follow-up shorter than 549 days have been excluded. ^e^ The uninfected subjects with a follow-up shorter than 731 days have been excluded. CI: Confidence interval.

**Table 2:** SARS-CoV-2 reinfections for the total sample and according to selected characteristics.

	Total Sample	Reinfections ^a^	Reinfection
Variables	*n*	% (*n*)	AHR (95% CI)
Overall	121,412	5.39 (6541)	--
Age class, years			
60+	28,761	5.49 (1579)	1 (ref.)
30–59	62,009	6.17 (3829)	1.45 (1.34–1.57)
10–29	30,642	3.70 (1133)	1.17 (1.07–1.28)
Mean age in years (SD)	46.1 ± 19.8	43.7 ± 18.1	1.00 (1.00–1.00)
Gender			
- Males	55,888	4.69 (2619)	1 (ref.)
- Females	65,524	5.99 (3922)	1.37 (1.30–1.44)
Severe COVID-19 after the first infection			
- No	118,125	5.22 (6171)	1 (ref.)
- Yes	3287	11.3 (370)	0.88 (0.79–0.98)
Diabetes ^b^			
- No	115,515	5.40 (6239)	1 (ref.)
- Yes	5897	5.12 (302)	1.04 (0.91–1.18)
Hypertension ^b^			
- No	106,711	5.50 (5871)	1 (ref.)
- Yes	14,701	4.56 (670)	1.15 (1.04–1.27)
Cardiovascular diseases ^b^			
- No	110,945	5.31 (5895)	1 (ref.)
- Yes	10,467	6.17 (646)	0.93 (0.85–1.03)
COPD ^b^			
- No	116,246	5.34 (6210)	1 (ref.)
- Yes	5166	6.41 (331)	1.14 (1.02–1.27)
Kidney diseases ^b^			
- No	119,339	5.36 (6400)	1 (ref.)
- Yes	2073	6.80 (141)	1.23 (1.03–1.47)
Cancer ^b^			
- No	115,008	5.43 (6247)	1 (ref.)
- Yes	6404	4.59 (294)	1.05 (0.93–1.19)
At least one chronic condition ^c^			
- No	93,920	5.39 (5062)	1 (ref.)
- Yes	27,492	5.38 (1479)	1.13 (1.05–1.20)
SARS-CoV-2 vaccine ^d^			
- No doses	15,030	11.2 (1681)	1 (ref.)
- 1 or 2 doses	38,741	8.14 (3154)	0.69 (0.65–0.74)
- 3 or more doses	67,641	2.52 (1706)	0.71 (0.66–0.76)

AHR: adjusted hazard ratio; CI: confidence interval; SD: standard deviation; ref.: reference category. ^a^ Two positive tests detected at least 45 days apart with at least one negative test detected between the first and second episode. ^b^ Please see text for details. ^c^ At least one among diabetes, hypertension, cardiovascular diseases, chronic obstructive pulmonary diseases, kidney diseases, or cancer. ^d^ Subjects receiving at least one dose of BNT162b2, ChAdOx1 nCoV-19, mRNA-1273, NVX-CoV2373, or JNJ-78436735 vaccines at least 14 days before reinfection.

## Data Availability

The data used for this study are available from the corresponding author upon reasonable request.

## Supplementary Materials

The following supporting information can be downloaded at: https://www.mdpi.com/article/10.3390/life13112111/s1, Table S1: SARS-CoV-2 reinfections, for the total sample and according to selected characteristics, during the pre-Omicron waves (from 2 March 2020 to 31 December 2021). Table S2: SARS-CoV-2 reinfections, for the total sample and according to selected characteristics, during the Omicron wave (from 1 January 2022).
