# Supplementary material for: Risk of SARS-CoV-2 Reinfection 3 Years after the Start of the Pandemic: A Population-Level Observational Study
Source: Life (Basel). 2023 Oct 24;13(11):2111. doi: 10.3390/life13112111 (PMC10672528; doi:10.3390/life13112111)
Supplement: Supplementary file 1 [file life-13-02111-s001.zip › life-2607426-supplementary.pdf]

Table S1. SARS-CoV-2 reinfections, for the total sample and according to selected characteristics, during the pre-Omicron waves (from March 2, 2020 to December 31, 2021). The following subjects were excluded from the overall sample: 104,989 subjects who had no positive swab before November 17, 2021 (to account for a minimum of 45 days between the first and second infection). Also, 2408 subjects who had a second positive swab after December 31, 2021 were reassigned to the group without a reinfection.

Variables	**Total sample** N	**Reinfections** [a] % (n)	**Reinfection** AHR (95% CI)
Overall	16,423	0.37 (60)	--
Age class, years			
60+	4172	0.70 (29)	1 (ref.)
30-59	8326	0.31 (26)	2.37 (0.79-7.07)
10-29	3925	0.13 (5)	5.21 (1.65-16.5)
Mean age in years (SD)	45.6±20.1	35.4±17.2	0.97 (0.96-0.99)
Gender			
- Males	8316	0.41 (34)	1 (ref.)
- Females	8107	0.32 (26)	1.34 (0.80-2.24)
Severe COVID-19 after the first infection			
- No	14,507	0.39 (57)	1 (ref.)
- Yes	1916	0.16 (3)	0.71 (0.21-2.44)
Diabetes [b]			
- No	15,367	0.38 (58)	1 (ref.)
- Yes	1056	0.19 (2)	1.17 (0.26-5.38)
Hypertension [b]			
- No	14,347	0.40 (57)	1 (ref.)
- Yes	2076	0.14 (3)	0.79 (0.20-3.20)
Cardiovascular diseases [b]			
- No	13,621	0.40 (54)	1 (ref.)
- Yes	2802	0.21 (6)	1.32 (0.48-3.65)
COPD [b]			
- No	15,372	0.37 (57)	1 (ref.)
- Yes	1051	0.29 (3)	0.99 (0.31-3.20)
Kidney diseases [b]			
- No	15,923	0.38 (60)	Not estimable
- Yes	500	0.00 (0)	Not estimable
Cancer [b]			
- No	15,581	0.37 (58)	1 (ref.)
- Yes	842	0.24 (2)	1.31 (0.31-5.61)
At least one chronic condition [c]			
- No	11,730	0.43 (50)	1 (ref.)
- Yes	4693	0.21 (10)	0.98 (0.46-2.10)
SARS-CoV-2 Vaccine [d]			

- No doses	13,025	0.42 (55)	1 (ref.)
- 1 or 2 doses	3285	0.15 (5)	0.39 (0.16-0.99)
- 3 or more doses	113	0.00 (0)	Not estimable

AHR: adjusted hazard ratio; CI: confidence interval; SD: standard deviation; ref.: reference category.

[a] Two positive tests detected at least 45 days apart with at least 1 negative test detected between the first and second episode.

[b] Please see text for details.

[c] At least one among diabetes, hypertension, cardiovascular diseases, chronic obstructive pulmonary diseases, kidney diseases or cancer.

[d] Subjects receiving at least one dose of BNT162b2, ChAdOx1 nCoV-19, mRNA-1273, NVX-CoV2373 or JNJ-78436735 vaccines, at least 14 days before the reinfection. Only the vaccinations that were performed before the Omicron wave (January 1, 2022) were considered in this analysis.

Table S2 SARS-CoV-2 reinfections, for the total sample and according to selected characteristics, during the Omicron wave (from January 1, 2022). The following subjects were excluded from the overall sample: 60 subjects who had a reinfection before January 1, 2022.

	Total sample	Reinfections [a]		Reinfection
Variables	N	% (n)	p [b]	AHR (95% CI)
Overall	121,352	5.34 (6481)	--	--
Age class, years				
60+	28,732	5.39 (1550)		1 (ref.)
30-59	61,983	6.14 (3803)	<0.001	1.45 (1.34-1.57)
10-29	30,637	3.68 (1128)	0.001	1.16 (1.06-1.27)
Mean age in years (SD)	46.1±19.8	43.7±18.1	0.4	1.00 (1.00-1.00)
Gender				
- Males	55,862	4.64 (2593)		1 (ref.)
- Females	65,490	5.94 (3888)	<0.001	1.37 (1.30-1.44)
Severe COVID-19 after the first infection				
- No	118,068	5.18 (6114)		1 (ref.)
- Yes	3284	11.2 (367)	0.026	0.88 (0.79-0.98)
Diabetes [c]				
- No	115,457	5.35 (6181)		1 (ref.)
- Yes	5895	5.09 (300)	0.6	1.04 (0.91-1.17)
Hypertension [c]				
- No	106,654	5.45 (5814)		1 (ref.)
- Yes	14,698	4.54 (667)	0.007	1.15 (1.04-1.27)
Cardiovascular diseases [c]				
- No	110,891	5.27 (5841)		1 (ref.)
- Yes	10,461	6.12 (640)	0.13	0.93 (0.84-1.02)
COPD [c]				
- No	116,189	5.30 (6153)		1 (ref.)
- Yes	5163	6.35 (328)	0.026	1.14 (1.02-1.27)
Kidney diseases [c]				
- No	119,279	5.32 (6340)		1 (ref.)
- Yes	2073	6.80 (141)	0.016	1.24 (1.04-1.48)
Cancer [c]				
- No	114,950	5.38 (6189)		1 (ref.)
- Yes	6402	4.56 (292)	0.4	1.05 (0.93-1.19)
At least one chronic condition [d]				
- No	93,870	5.34 (5012)		1 (ref.)
- Yes	27,482	5.35 (1469)	<0.001	1.13 (1.05-1.20)
SARS-CoV-2 Vaccine [e]				
- No doses	14,977	10.9 (1628)		1 (ref.)
- 1 or 2 doses	38,734	8.12 (3147)	<0.001	0.71 (0.67-0.76)

- 3 or more doses	67,641	2.52 (1706)	<0.001	0.73 (0.68-0.78)

AHR: adjusted hazard ratio; CI: confidence interval; SD: standard deviation; ref.: reference category.

[a] Two positive tests detected at least 45 days apart with at least 1 negative test detected between the first and second episode.

[b] p-value from the Cox proportional hazard model.

[c] Please see text for details.

[d] At least one among diabetes, hypertension, cardiovascular diseases, chronic obstructive pulmonary diseases, kidney diseases or cancer.

[e] Subjects receiving at least one dose of BNT162b2, ChAdOx1 nCoV-19, mRNA-1273, NVX-CoV2373 or JNJ-78436735 vaccines, at least 14 days before the reinfection.
